# Concentration of interleukin 17A and measurement properties of the neutrophiles with food allergy hypersensitivity patients

**DOI:** 10.1186/2045-7022-3-S3-P104

**Published:** 2013-07-25

**Authors:** M Żbikowska-Gotz, K Pałgan, E Gawrońska-Ukleja, A Kuźmiński, M Przybyszewski, Z Bartuzi

**Affiliations:** 1 Department of Allergology, Clinical Immunology and Internal Medicine Collegium Medicum in Bydgoszcz, Nicolaus Copernicus University in Toruń, Bydgoszcz, Poland

## Background

IL17 is a cytokine produced by activated helper lymphocytes Th17. It plays a role in the mechanisms of allergic reactions involving neutrophils through the induction of poduction cytokines and chemokines. Affects the activation, recruitment and migration of neutrophils into the tissue by regulating inflammatory reaction. Activated neutrophils secrete elastase and ROS (reactive oxygen derivatives) important mediators of the inflammatory process responsible for tissue damage.

## Methods

Analyses: Interleukin 17A concentrations in serum, neutrophil elastase concentration in serum, ROS production by unstimulated neutrophils, the relation between serum levels of IL17A and metabolic activity of neutrophils measured by chemiluminescence assay, and serum elastase.

The study included 30 patients with food allergy diagnosed based on history, clinical signs, the presence of positive SPT and serum specific IgE against food allergens selected immunoenzymatic method using sets of Hycor. The control group consisted of 10 healthy volunteers. Concentration was determined by ELISA using IL17A sets eBioscience and elastase with sets BENDERMED SYSTEMS. Examine chemiluminescence of unstimulated neutrophil by kinetic method luminolo-dependant during 40 minutes with a luminometer LUMINOSCAN - Labsystem.

## Results

Serum concentrations of IL17A and chemiluminescence values obtained by unactivated neutrophils and elastase levels were higher in patients with allergic hypersensitivity to food than in the group of healthy subjects.

## Conclusion

The results obtained confirm the role and increased neutrophiles activity in allergy inflamatory process with examined patients.

## Disclosure of interest

None declared.

